# Proteomic Characterization of Host-Pathogen Interactions during Bovine Trophoblast Cell Line Infection by *Neospora caninum*

**DOI:** 10.3390/pathogens9090749

**Published:** 2020-09-15

**Authors:** Javier Regidor-Cerrillo, Dong Xia, Laura Jiménez-Pelayo, Marta García-Sánchez, Esther Collantes-Fernández, Nadine Randle, Jonathan Wastling, Luis-Miguel Ortega-Mora, Pilar Horcajo

**Affiliations:** 1 SALUVET-Innova, Faculty of Veterinary Sciences, Complutense University of Madrid, Avda. Puerta de Hierro s/n, 28040 Madrid, Spain; jregidor.saluvetinnova@gmail.com; 2 Department of Comparative Biomedical Sciences, Royal Veterinary College, University of London, Royal College Street, London NW1 0TU, UK; dxia@rvc.ac.uk; 3 SALUVET, Animal Health Department, Faculty of Veterinary Sciences, Complutense University of Madrid, Ciudad Universitaria s/n, 28040 Madrid, Spain; ljpelayo@ucm.es (L.J.-P.); martag17@ucm.es (M.G.-S.); esthercf@ucm.es (E.C.-F.); luis.ortega@ucm.es (L.-M.O.-M.); 4 Department of Infection Biology, Institute of Infection & Global Health, University of Liverpool, Liverpool Science Park IC2, 146 Brownlow Hill, Liverpool L3 5RF, UK; Nadine.Randle@liverpool.ac.uk; 5 Faculty of Natural Sciences, Keele University, Keele, Staffordshire ST5 5BG, UK; j.wastling@keele.ac.uk

**Keywords:** *Neospora caninum*, proteome, isolate virulence, bovine trophoblast cell line, host–parasite interaction

## Abstract

Despite the importance of bovine neosporosis, relevant knowledge gaps remain concerning the pathogenic mechanisms of *Neospora caninum*. Infection of the placenta is a crucial event in the pathogenesis of the disease; however, very little is known about the relation of the parasite with this target organ. Recent studies have shown that isolates with important variations in virulence also show different interactions with the bovine trophoblast cell line F3 in terms of proliferative capacity and transcriptome host cell modulation. Herein, we used the same model of infection to study the interaction of *Neospora* with these target cells at the proteomic level using LC-MS/MS over the course of the parasite lytic cycle. We also analysed the proteome differences between high- (Nc-Spain7) and low-virulence (Nc-Spain1H) isolates. The results showed that mitochondrial processes and metabolism were the main points of *Neospora*-host interactions. Interestingly, Nc-Spain1H infection showed a higher level of influence on the host cell proteome than Nc-Spain7 infection.

## 1. Introduction

*Neospora caninum*, a protozoan parasite, is the causative agent of neosporosis, one of the most important infectious causes of abortion in cattle. Currently, no vaccine or therapy is available for the prevention or treatment of neosporosis, despite this disease being responsible for important economic losses to the cattle industry worldwide [1,2].

The major route of *N. caninum* infection is transplacental from dams to their foetuses after primary infection of dams during pregnancy or after recrudescence of the infection in persistently infected dams. Abortion or congenital infection occurs when parasites multiply, cross the placenta and infect the foetus. In addition to damage in the placenta and foetal tissues due to parasite multiplication, the inflammatory immune response (biased to Th1) triggered in the placenta after *N. caninum* infection is associated with abortion [3]. Placental infection is therefore a crucial event in the pathogenesis of bovine neosporosis.

Recently, the bovine placental trophoblast cell line F3 has been proposed as a good in vitro model for studying the interaction of *N. caninum* with its target cells [4]. Using this model, we have previously shown that *N. caninum* infection modulates the transcriptome of host cells, especially in pathways such as extracellular matrix organization and cholesterol metabolism, favouring a pro-inflammatory response. In addition, some differences in parasite–trophoblast interactions between isolates have been described [5,6]. Parasite intraspecific variation in virulence has been widely demonstrated, and isolates have been characterized with low (e.g., Nc-Spain1H), moderate (e.g., Nc-1) and high virulence (e.g., Nc-Liv, Nc-Spain7) according to their capacity to produce foetal death after infection of dams during pregnancy [7,8,9,10,11].

In this work, we used a proteomic approach to investigate the interaction of *N. caninum* with a bovine trophoblast cell line throughout the tachyzoite lytic cycle (invasion, multiplication and egress). In addition, we included two isolates with large differences in virulence and in their in vitro behaviour to gain knowledge of factors that could be determinants of virulence. The results revealed mitochondrial processes and metabolism as the main interface of *Neospora*-host interactions. Interestingly, infection with the low-virulence isolate Nc-Spain1H showed a higher impact on the host cell proteome than that with Nc-Spain7. This finding needs to be further investigated, as it could reveal a strategy by which a highly virulent isolate better establishes infection, which goes unnoticed if it does not overly disrupt the host cells, or a modulation of a low-virulence isolate aimed at avoiding host cell death and achieving more efficient transmission.

## 2. Results and Discussion

### 2.1. Neospora caninum and Bovine Trophoblast Cell Proteomes

In total, 1192 bovine proteins were identified and quantified. This number of identified and quantified proteins is comparable to that in other studies in trophoblastic cell lines [12]. We also identified and quantified 334 *N. caninum* proteins. Although we have identified fewer proteins than in previous studies dedicated to purified parasite proteomes [13,14,15,16,17], to our knowledge, this is the first time the host and *Neospora* proteomes have been studied simultaneously. 

### 2.2. Host Proteome Remodelling during N. caninum Infection 

**Figure 1:**
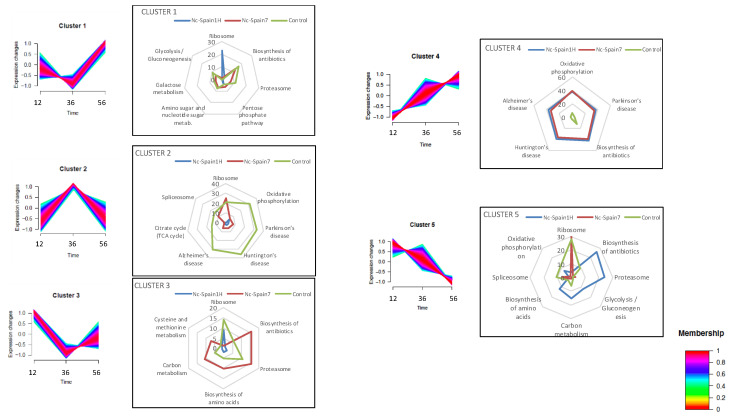
Clusters of host protein expression patterns reveal various profiles of enriched biological processes. Five clusters of expression patterns are shown, where profiles of the main enriched pathways in each cluster and the number of proteins associated with each condition are summarized in the radar charts: Nc-Spain1H-infected cells (blue), Nc-Spain7-infected cells (red) and uninfected cells (green).

To analyse the influence of *N. caninum* infection on the host cell proteome, we investigated the differences in the protein kinetics among F3 cells infected with both isolates and uninfected cells throughout the tachyzoite lytic cycle (after invasion 12 h proliferation 36 h and egress 56 h). Patterns of quantified proteins in each condition were categorized into five clusters based on similarity of protein abundance kinetics through the course of *N. caninum* infection (Figure 1 and Table S1).

The main variable processes throughout the tachyzoite lytic cycle were related to protein synthesis and turnover (“ribosome” and “proteasome”) and to metabolism: “carbon metabolism”, “glycolysis/gluconeogenesis”, “pentose phosphate pathway”, “amino sugar and nucleotide sugar metabolism”, “cysteine and methionine metabolism”, and “biosynthesis of antibiotics”. These findings showed host metabolism was highly regulated by parasite infection, mainly with the Nc-Spain1H isolate. This result has been previously shown for this and other parasites [5,18,19,20], and host metabolic events are important for establishment of infection, initiating host immunity and determining the eventual success of infection; therefore, it would be worth deepening knowledge of the types of *Neospora* host-metabolism regulation.

The profiles of clusters one and three are “V” shaped, with the lowest protein abundance during active multiplication at 36 h post-infection (hpi), although cluster one has lower starting expression values and higher values at 56 hpi, and cluster three is the opposite. In both cases, the Nc-Spain1H-infected cells showed only “ribosome pathway” as the over-represented process, suggesting inhibition of protein synthesis during active multiplication of the parasite that is not observed in Nc-Spain7 infection. Uninfected cells also showed inhibition, according to cluster three. Interestingly, cells infected with the high-virulence isolate had a very similar profile to that of uninfected cells in cluster one and cluster five. Cluster five differed from the previous results mainly at 56 hpi because in this cluster there is a decrease in the abundance from invasion to egress (Figure 1). In Nc-Spain1H-infected cells, processes such as “proteasome”, “carbon metabolism”, and “glycolysis/gluconeogenesis” have this profile of inhibition as parasite multiplication increases. In the other two groups, these processes have a cluster one profile, with the highest protein abundance at 56 hpi. “Ribosome pathway” was the main enriched process among proteins of cluster five in control and Nc-Spain7-infected cells. The greater similarity of proteome profiles between Nc-Spain7-infected cells and uninfected cells could indicate a greater host response against the low-virulence isolate, which could slow parasite multiplication and dissemination. Similar results have been found in transcriptomic analyses of infected trophoblast [5] and macrophage [21] cells and in analyses of gene expression of placentomes from an experimental infection in bovines, which could suggest a higher capacity of high-virulence isolates to restrain the host response at early stages of infection [22]. 

The main differences between infected and uninfected cells were found in clusters two and four. These clusters are characterized by an increase in protein abundance during parasite multiplication; however, in cluster two there is a decrease during egress (56 hpi) that does not happen in cluster four because the abundance continues increasing. In this case, the cells infected by both isolates clearly differ from uninfected cells. Pathways related to diseases (“Parkinson”, “Huntington”, and “Alzheimer”) and metabolism energy (Additional file one) had the profile of cluster two in uninfected cells and cluster four in cells infected with both isolates. Most of these pathways are clearly related to mitochondrial function, as mitochondrial dysfunction plays an essential role in neurodegenerative diseases [23], and the citrate cycle and oxidative phosphorylation are processes that take place within mitochondria. Proteins of all mitochondrial respiratory chain complexes (NADH-ubiquinone oxidoreductase, ubiquinone-cytochrome c oxidoreductase, cytochrome c oxidase, succinate dehydrogenase and ATP synthase) and the citrate cycle (citrate synthase, isocitrate dehydrogenase, malate dehydrogenase, succinate dehydrogenase and succinate-CoA ligase) showed different profiles between infected and uninfected cells, and the continuous increase in the abundance of these proteins in infected cells could indicate a high energy demand. Perturbation of host mitochondrial processes following *N. caninum* and *Toxoplasma gondii* infection has been previously shown [24,25,26,27,28,29]. In addition, in *T. gondii* it has been suggested that host mitochondrial regulation is strain-specific [26]; however, in this study, the profile of host mitochondrial proteins is similar in cells infected with both isolates, and protein abundance increased in relation to parasite multiplication (cluster four), so regulation could not depend on the isolate. The mitochondrial respiratory chain is a major source of reactive oxygen species (ROS), products of an important host immune response against *N. caninum* infection. The profile of these proteins increases according to parasite multiplication and can reflect the defence mechanism of trophoblast cells against infection. In a previous study, we showed that *Neospora* is able to repress ROS production in bovine macrophages, showing greater ability with the high-virulence isolate Nc-Spain7 [30]. The pentose phosphate pathway is a predominant producer of cellular NADPH and is thus critical for antioxidant defence. This pathway appeared to be modulated exclusively during infection with Nc-Spain7 (cluster 1, Table S1), which could mean that the parasite modulates this route to reduce ROS levels and facilitate its multiplication. 

We also carried out additional differential expression analyses between the three conditions at the three time points to achieve higher resolution of the host–proteome modulation by *N. caninum* (Table S2). The results support the main findings described previously. Very few differences were found between conditions in the earliest stages of infection (12 and 36 hpi), with the exception of some mitochondrial proteins. This finding supports the early relationship between *Neospora* and this host organelle, which has been shown previously in *Neospora* and in *T. gondii* [24,25,27]. 

At the latest stage of infection, 195 proteins were differentially abundant in the comparison of Nc-Spain1H-infected cells vs. uninfected cells and only 41 in the comparison of Nc-Spain7-infected cells vs. uninfected cells, which reflects the ability of Nc-Spain7 to not disrupt host cells too much. In Nc-Spain1H-infected cells, the altered processes were those described previously, with a key role in metabolic regulation, which has been previously associated with host proteome modulation by intracellular *T. gondii* multiplication [25,31] and is related to many other cellular functions, such as energetics, cell cycle and cell death [25]. In Nc-Spain7 infections, it is worth noting that most of the regulated proteins with higher abundance in infected cells were associated with the “oxidation–reduction process”, perhaps as a survival strategy of the high-virulence isolate, as mentioned before.

Between isolates, only 12 proteins were differentially abundant, so we could conclude similar host modulation by both isolates, as previously suggested [5].

### 2.3. Host Immune Response during N. caninum Infection

Trophoblast cells are a recognized component of the immune system and are capable of responding to pathogens [32,33]. Recently, it has been shown by quantitative PCR analyses that trophoblast cells are able to respond to *Neospora* infection with a pro-inflammatory cytokine expression profile [6]. In the present study, none of these immune–related proteins were identified. Therefore, we investigated whether other immune-related proteins had a different abundance profile between conditions. We reviewed proteins involved in the immune response based on Gene Ontology (GO) and pathway annotations, and out of ten quantified proteins in our datasets, nine of them exhibited differential expression profiles between conditions (Table S3). More remarkably, differences were found between infected and uninfected cells. It is worth noting the proteins CD166 antigen and Presenilin-1 are highly inhibited at the early stage of infection. The CD166 antigen, or Activated Leukocyte Cell Adhesion Molecule (ALCAM), is implicated in the host immune response, e.g., cell adhesion interactions of activated T and B cells during inflammatory infection [34,35], so its early inhibition could facilitate infection establishment. By contrast, Beta-2-microglobulin, Complement component 1 Q subcomponent-binding protein and 60 kDa heat shock protein are multifunctional proteins, but all of them are involved in inflammation and infection processes. These proteins increased in abundance in infected cells in relation to tachyzoite multiplication. The results showed certain immune responses of trophoblast cells to infection. However, perhaps due to a lack of stimulation of other immune cells or even due to the limitations of the analysis technique, we were not able to find key elements such as cytokines widely studied by other authors in placental cells [6,36,37,38]. No clear differences were found between isolates, corroborating the results previously found by gene expression analyses in the same model of *N. caninum* [6].

### 2.4. Proteomic Differences between High- and Low-Virulence N. caninum Isolates at Different Stages of the Lytic Cycle

Of the 334 *N. caninum* proteins quantified, only 8, 1 and 19 had differential abundances between the isolates at 8, 36 and 56 hpi, respectively. These results, in terms of the number and identity of the proteins, did not agree with previous results comparing these isolates [15,17]. However, use of this model to study the host and *Neospora* proteomes simultaneously, without parasite purification, could limit the study of the parasite, as only those proteins with high abundances will be detected. As expected from previous results, a low correlation was found with the transcriptome results obtained previously in a study with a similar design [5].

Among our results, it is worth noting that some rhoptry proteins were observed. Rhoptry proteins are recognized among the major virulence factors and effectors for host modulation in *T. gondii* [39,40], and some of them have also been described as virulence factors in *N. caninum* [41,42]. The high-virulence isolate Nc-Spain7 showed an increased abundance at 8 hpi of a predicted member of the rhoptry kinase family, ROP20, specific for *N. caninum* (NCLIV_069590), which is orthologous but not syntenic to the *T. gondii* virulence factor ROP24, and higher abundance of another predicted member of the rhoptry kinase family, ROP20, specific for *N. caninum* (NCLIV_068850), which has been consistently found with higher protein abundance and mRNA expression in previous studies [5,17,43]. In addition, two other predicted rhoptry proteins (NCLIV_011700, NCLIV_031550) were more abundant in the low-virulence isolate at 56 hpi. A dense granule protein (NCLIV_041120), another key effector in host modulation [44], was also more abundant in the Nc-Spain1H isolate. Additional studies would be very useful to elucidate the role of these proteins in *N. caninum* infection.

The rest of the differentially abundant proteins were mainly ribosomal proteins and proteins related to metabolism. These processes are consistently found to be differentially regulated between isolates [5,17,43,45]. Investigating this regulation in depth may give interesting information, as these processes previously have been related to virulence in other parasites [46,47]. In addition, we would like to point out that the genome of this parasite is still under study and ongoing work suggested the possibility of an improved annotated genome in future that could influence specific parts of the results obtained in this section.

## 3. Materials and Methods 

### 3.1. Parasites and Cell Cultures

Nc-Spain7 and Nc-Spain1H isolates were maintained in the MARC-145 cell line cultured in Dulbecco’s modified Eagle’s medium (DMEM, Sigma-Aldrich, St. Louis, MO, USA) supplemented with 10% foetal bovine serum (FCS), penicillin (100 U/mL) and streptomycin (100 μg/mL) (Gibco BRL, Paisley, UK) in 5% CO_2_/37 °C [48]. FBS was previously checked for the absence of anti-*Neospora* IgG by indirect fluorescent antibody test (IFAT).

The bovine trophoblast cell line F3, provided by Dr. Pfarrer (University of Veterinary Medicine Hannover, Hannover, Germany) [49], was maintained in DMEM/Ham’s F12 containing 10% FCS, 100 IU/mL penicillin, 100 mg/mL streptomycin and 2 mM glutamine. All experiments were developed with cells below 27 passages, while the cell lines maintained all of their features [49,50]. 

### 3.2. Experimental Design and Sample Production for Proteome Analyses

F3 infections for proteome analyses were performed as described previously [4]. Briefly, F3 monolayers in DMEM free of phenol red (Gibco, Gaithersburg, MD, USA) and FBS were inoculated with purified tachyzoites at an MOI of 8 for Nc-Spain7 and 10 for Nc-Spain1H in order to obtain the highest number of infected cells with a similar infection rate for both isolates [4]. Additional flasks with F3 monolayers were maintained under the same conditions but were not infected, as controls. Infected and non-infected cells were washed 3 times with phosphate-buffered saline (PBS) at 4 h post-infection (hpi) to remove non-invading tachyzoites. Cells were recovered at 12 hpi (after invasion but prior to tachyzoite duplication), at 36 hpi (in active proliferation) and at 56 hpi (early egress) from T75 cm^2^ flasks by scraping cells in 5 mL of PBS supplemented with protease and phosphatase inhibitor cocktail (Sigma-Aldrich, St. Louis, MO, USA). Cells were pelleted by centrifugation at 1350× *g* for 10 min and stored at −80 °C until proteome analysis. All experiments involved three biological replicates obtained in three different experiments.

### 3.3. LC-MS/MS Analyses

All analyses were essentially performed as previously described [17]. Pellets were resuspended in 25 mM ammonium bicarbonate and RapiGestTM (Waters MS Technologies, Milford, MA, USA) for protein solubilization, reduced with DTT and alkylated with iodoacetamide for trypsin digestion. Then, the digests were analysed using an LC-MS/MS system comprising an Ultimate 3000 nano system coupled to a Q-Exactive mass spectrometer (Thermo Fisher Scientific, Waltham, MA, USA). Reversed-phase liquid chromatography was performed using an Ultimate 3000 nanosystem with a linear gradient of 5–40% Buffer B (80% acetonitrile in 0.1% formic acid) in 0.1% formic acid (Buffer A). The Q-Exactive was operated in data-dependent mode with survey scans acquired at a resolution of 70,000 at m/z 200. Up to the top 10 most abundant isotope patterns were selected and fragmented by higher energy collisional dissociation with normalized collision energies of 30. The maximum ion injection times for the survey scan and the MS/MS scans were 250 and 100 ms, respectively.

### 3.4. Data Analyses 

For proteome data analyses, Thermo RAW files were imported into Progenesis QI (version 2.0, Nonlinear Dynamics, Durham, CA, USA). Replicate runs were time aligned using default settings and an auto-selected run as a reference. Spectral data were transformed into mgf files with Progenesis QI and exported for peptide identification using the Mascot (version 2.3, Matrix Science, London, UK) search engine, the *Bos taurus* database (SwissProt release Feb 2015) and ToxoDB-13_Ncaninum LIV_Annotated Proteins (version 13, ToxoDB), the current *Neospora* genome annotation. Personal communications suggested a project aiming to improve annotation is currently ongoing, which can be searched against with the raw data deposited on ProteomeXchange in future. Finally, protein abundance (iBAQ) was calculated as the sum of all the peak intensities (from Progenesis output) divided by the number of theoretically observable tryptic peptides for a given protein and clustered by fuzzy c-means using GProX [51]. One way ANOVA was used for differential expression analysis, which was implemented in Progenesis (http://www.nonlinear.com/progenesis/qi-for-proteomics/v1.0/faq/experiment-design.aspx).

The mass spectrometry proteomics data have been deposited in the ProteomeXchange Consortium via the PRIDE [52] partner repository with the dataset identifier PXD019354.

The parasite-identified proteins were classified according to the GO terms (annotated and predicted) on the ToxoDB website [19] for the Nc-Liverpool isolate (ToxoDB-13_NcaninumLIV_AnnotatedProteins), *T. gondii* syntenic homologues (version 13, ToxoDB) and previous reports [5,17,53,54]. 

## 4. Conclusions

While efforts have been focused on determining virulence factors in *N. caninum* [41,42,55] and transcriptomic or proteomic differences between isolates [15,17], less attention has been paid to host–pathogen interactions, which are essential for a holistic understanding of infection biology and disease processes. In this study, for the first time, the proteomes of bovine trophoblast cells (target cells for *N. caninum*) infected with two well-differentiated isolates, the high-virulence Nc-Spain7 and the low-virulence Nc-Spain1H isolates, were studied at different time points of the tachyzoite lytic cycle post infection. Mitochondrial processes and metabolism were the main points of focus of *Neospora*–host interactions. Interestingly, Nc-Spain1H infection showed a greater influence on the host cell proteome than Nc-Spain7 infection. These results confirm previously observed host modulation events by both isolates but with a greater host response against the low-virulence isolate or greater modulation by this isolate, perhaps more adapted to avoid abortion and allow its transmission. A specific immune response against the infection was not found in this study. This result may highlight the need to perform these types of studies in models that better mimic the real infection environment. 

## Supplementary Materials

The following are available online at https://www.mdpi.com/2076-0817/9/9/749/s1, Table S1: Pathway enrichment analyses of host protein clusters; Table S2: Differentially abundant host proteins between isolates at 12, 36 and 56 h post infection; and Table S3: Immune-related proteins.
